# Variants in the IL7RA gene confer susceptibility to multiple sclerosis in Caucasians: evidence based on 9734 cases and 10436 controls

**DOI:** 10.1038/s41598-017-01345-8

**Published:** 2017-04-26

**Authors:** Hong Liu, Jian Huang, Mengmeng Dou, Yong Liu, Biying Xiao, Xu Liu, Zunnan Huang

**Affiliations:** 1 Key Laboratory for Medical Molecular Diagnostics of Guangdong Province, Dongguan Scientific Research Center, Guangdong Medical University, Dongguan, Guangdong 523808 China; 2 School of Pharmacy, Guangdong Medical University, Dongguan, Guangdong 523808 China; 3 Department of Neurosurgery, Dalingshan Hospital, Dongguan, Guangdong 523819 China; 4 Key Laboratory for Research and Development of Natural Drugs of Guangdong Province, Zhanjiang, Guangdong 524023 China; 5 The Second School of Clinical Medicine, Guangdong Medical University, Dongguan, Guangdong 523808 China

## Abstract

Recently, numerous genome wide association studies (GWAS) and other case-control association studies examining the relationship between interleukin-7 receptor α chain (IL7RA) gene rs3194051, rs987107, rs11567686, and rs11567685 variants and multiple sclerosis (MS) risk have been conducted, but the conclusions have been inconsistent. The main objective of this meta-analysis was to more precisely explore the association of these four IL7RA variants with MS development. Twenty-seven eligible studies involving 9734 cases and 10436 controls were included in the present meta-analysis. Power calculation, publication bias, sensitivity analysis and cumulative meta-analysis were performed to derive a reliable conclusion. Our study indicated three IL7RA loci were significantly associated with increasing MS risk (rs3194051: recessive model: OR = 1.22, 95% CI 1.08–1.38; rs987107: recessive model: OR = 1.44, 95% CI 1.22–1.69; and rs11567686: dominant model: OR = 1.18, 95% CI 1.01–1.37). Additionally, IL7RA rs11567685 variants might not be related to MS development. In all, IL7RA locus polymorphisms could play an important role in the predisposition to MS, which could contribute to a better understanding the pathogenesis of multiple sclerosis.

## Introduction

Multiple sclerosis (MS), an inflammatory autoimmune disease of the central nervous system (CNS), is characterized by lymphocytic infiltration, demyelination and axonal loss^1^. It is estimated that MS affects approximately 2.5 million people throughout the world^2^, and approximately 400,000 Americans are currently diagnosed with MS, with 200 newly diagnosed cases each week^3^. Patients in the advanced stages of MS may have various neurological symptoms including ataxia, gait instability and cognitive deficits^4^, which seriously reduces the quality of their lives. Multiple sclerosis causes a heavy economic burden on society; for example, in 2009 the annual treatment cost for each patient was more than $23,000 in the United States^5^.

Although the exact etiology of MS is still not completely understood, there is growing evidence that the interplay between environmental factors including Epstein-Barr virus (EBV)^6^, latitude^7^, smoking^8^, and vitamin D^9^, and genetic factors contribute to the risk of developing MS^10^. In addition, it has been well established that variants in the major histocompatibility complex (MHC) gene on chromosome 6p21 are an extremely important genetic factor for MS susceptibility^11–13^. However, recent independent genome wide association studies (GWAS) have revealed some non-MHC MS susceptibility genes, such as CXCR5^14^, BCL10^15^, IL2RA^16^, IL7RA^17^ and CD86^18^.

The IL7RA gene is located on chromosome 5p13.2 and encodes the interleukin 7 receptor-α (namely CD127) protein, which plays a vital role in V(D)J recombination during lymphocyte development^19^ and controls the T lymphocyte receptor-γ loci approachability by histone acetylation and STAT5^20^. Over the last decade it has been established that this gene influences MS risk in Caucasians^21–23^. For example, in 2003, Teutsch *et al*. first identified the IL7RA rs11567686 and rs11567685 polymorphisms, which were suggested to have a potential association with susceptibility to MS^24^. In 2005, Zhang *et al*. described a significant association between IL7RA rs3194051 and rs987107 variants and an increased risk of MS observed in Swedish patients^23^. Subsequently, multiple studies were conducted to explore the impact of these IL7RA polymorphisms on the development and pathogenesis of MS in different ancestral groups; however, these studies provided conflicting results^17, 21, 22, 25–27^. To the best of our knowledge, no systematic review of such association has been carried out to contend with the issue of inconsistencies from different research studies. Therefore, we synthesized available evidence from all published studies regarding the relationship between IL7RA polymorphisms and MS and performed a meta-analysis to elucidate the association between these four single nucleotide polymorphisms (SNPs) and susceptibility to MS in Caucasians.

## Materials and Methods

### Search strategies

**Figure 1:**
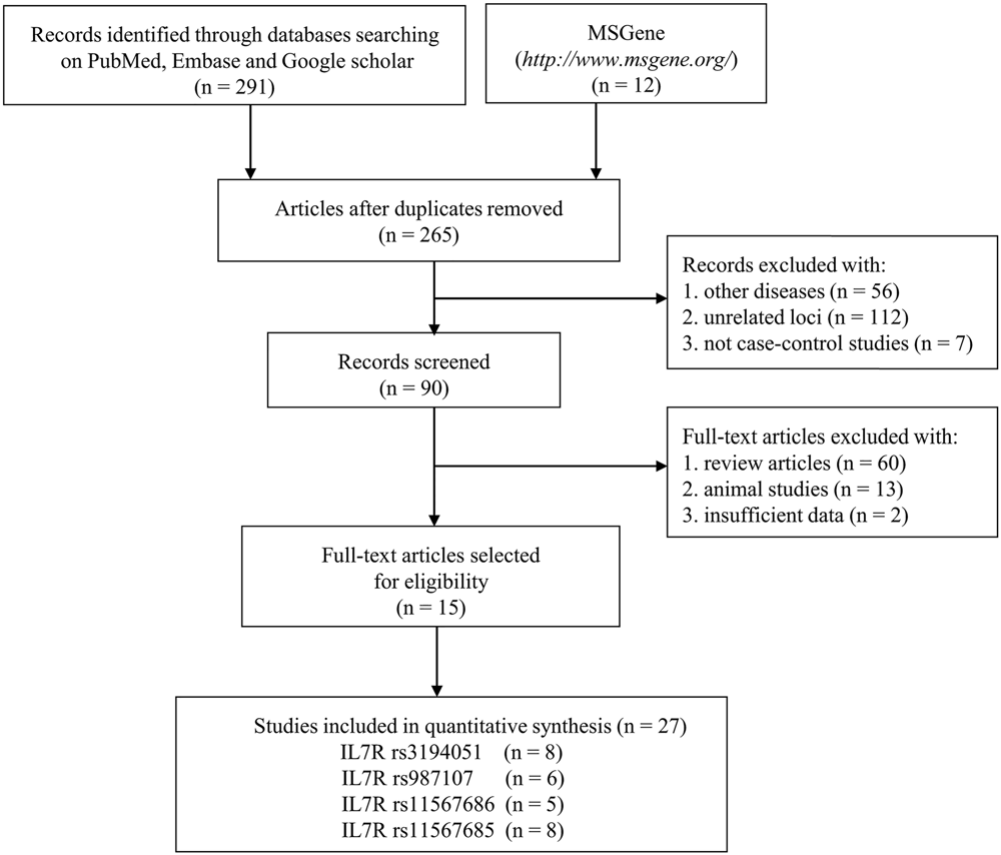
Flow diagram of the process used to select eligible studies.

Two reviewers systematically searched literature from the PubMed, Embase, Google Scholar, China National Knowledge Infrastructure (CNKI) and MS Gene (http://www.msgene.org/) databases (up to June 14, 2016). We first explored the CNKI database, but no eligible studies could be retrieved (data not shown in Fig. 1). We then performed a search of English databases using the following keywords: (interleukin 7 receptor OR IL7R OR ILRA OR IL-7R-alpha OR CDW127 OR IL7RA OR CD127) AND (polymorphism OR mutation OR variant) AND “multiple sclerosis”. Additional studies were manually examined from the references cited in the original literature. For case-control studies with overlapping data, the one with the largest sample size was included in this meta-analysis.

### Study inclusion criteria

The following inclusion criteria were used for selecting suitable studies: (i) the study was on the association of IL7R rs3194051, rs987107, rs11567686 or rs11567685 polymorphisms with MS; (ii) the study used a case–control design; (iii) the study provided raw genotype data (such as GG, GA and AA genotypes) for calculating the odds radio with 95% confidence interval. Articles that did not meet the above inclusion criteria were excluded from our meta-analysis.

### Data extraction

For each eligible study, the following data were extracted: (1) first author’s name and publication year, (2) area and ethnicity of the participants, (3) the number of cases and controls or the distribution of genotypes (4) source of control and genotyping method, (5) age and gender information, and (6) type and diagnostic criteria for multiple sclerosis. Two reviewers independently completed this step and collected the data carefully. Any disagreement was resolved by a discussion with a third reviewer.

### Quality assessment

The Newcastle-Ottawa Scale (NOS) criteria were used to assess the quality of retrieved studies, which included three aspects: object selection, comparability and exposure assessment^28^. Studies with at least six points were considered high quality studies.

### Statistical analysis

The Hardy-Weinberg equilibrium (HWE) was performed for testing the genotype distribution of the control group within each included study, and a *P*-value greater than 0.05 meant that the study sample was representative of the population in the corresponding area.

The pooled odds ratios (ORs) with 95% confidence intervals (CIs) were calculated to evaluate the strength of the relationship between each IL7RA polymorphism and MS risk. The estimated ORs were as follows:

- For rs3194051 SNP: GG vs. AA (OR1), GA vs. AA (OR2), and GG vs. GA (OR3).
- For rs987107 SNP: TT vs. CC (OR1), TC vs. CC (OR2), and TT vs. TC (OR3).
- For rs11567686 SNP: GG vs. AA (OR1), GA vs. AA (OR2), and GG vs. GA (OR3).
- For rs11567685 SNP: CC vs. TT (OR1), CT vs. TT (OR2), and CC vs. CT (OR3).

The above crude ORs were then used to determine the most applicable or ideal genetic model using the following method initially described by Thakkinstian *et al*.^29^ (Table 1):

1. If OR1 = +, OR2 = +, and OR3 = −, then a dominant model is suggested.
2. If OR1 = +, OR2 = −, and OR3 = +, then a recessive model is suggested.
3. If OR1 = −, OR2 = +, OR3 = +, and OR2 is at a lesser risk than both OR1 and OR3, then a complete overdominant model is suggested.
4. If OR1 = + +, OR2 +, and OR3 = +, then a codominant model is suggested.

**Table 1:** Multiple comparisons of genotype effects and possible modes of inheritance.

Mode of inheritance	MM vs. WW	MW vs. WW	MM vs. MW
	(OR1)*	(OR2)	(OR3)
Dominant	**+**	**+**	**−**
Recessive	**+**	**−**	**+**
Complete overdominant^#^	**−**	**+**	**+**
Codominant	**++**	**+**	**+**

Note: MM: homozygous mutant genotype; MW: heterozygous mutant genotype; WW: wild genotype.

*ORi is pooled odds ratio, complete overdominant model: OR2 is at a lesser risk than both OR1 and OR3. +means a significant result; + + means the effect size is greater than +; − means a non-significant result.

(+means a statistically significant result; + + means the effect size is greater than +; − means a non-significant result)

A *P*-value of less than 0.05 for the Z-test indicated statistical significance of the pooled ORs. We used the statistical software Power and Sample Size Calculation (PS) version 3.1.2 (http://biostat.mc.vanderbilt.edu/wiki/Main/PowerSampleSize)^30^ to perform power calculations regarding the association of each IL7RA variant with MS under the appropriate model, and a power value greater than 0.8 meant high statistical power in this meta-analysis. The *I* ^*2*^ test was conducted to estimate the between-study heterogeneity. The random-effect model (DerSimonian and Laird method) was used when significant heterogeneity existed among the studies (*I* ^*2*^ ≥ 50%); otherwise, the fixed-effect model (Mantel-Haenszel) was utilized. Sensitivity analysis was conducted by removing one study at a time, especially the one not in HWE, to evaluate the stability of the overall results. Additionally, a cumulative meta-analysis sorted upon sample size was carried out to explain precisely the association of IL7RA rs3194051, rs987107 and rs11567686 polymorphisms with MS risk under their special genetic models, which updated the pooled results every time a new study sample was added. Publication bias was estimated by Begg’s funnel plot and Egger’s tests among the eligible studies, and a *P*-value > 0.05 suggested no apparent bias. The software STATA 14.0 (Stata Corporation College Station, Texas, USA) was used for all statistical analyses.

## Results

### Literature search results

Figure 1 describes the procedure of the literature search and the study selection in this meta-analysis. Following the initial search strategy, 303 potential publications were identified. Among them, 38 were found to be duplicates. After these duplicates were removed, we obtained 265 articles, which included 56 that were related to other diseases, 112 concerning unrelated loci and 7 articles whose studies did not utilize a case-control design. Therefore, these 175 articles were excluded and 90 articles remained. Subsequently, we further screened these remaining articles (n = 90) by removing the reviews (n = 60), animal studies (n = 13) and those studies with missing partial genotype data (n = 2). Finally, 15 articles including 27 eligible studies were included in this meta-analysis^17, 21–27, 31–37^. Among these 27 individual studies, 8, 6, 5, and 8 studies were linked to IL7RA rs3194051, rs987107, rs11567686, and rs11567685 polymorphisms, respectively.

### Main characteristics of included studies

Table 2 provides the number of cases and controls, the number of genotypes of IL7RA rs3194051, rs987107, rs11567686, and rs11567685 loci, HWE and power analysis for each included study. From this table, it can be determined that the genotypic frequency distribution of the control group was consistent with HWE in all eligible studies, except for three (one reported by Zhang *et al*.^23^ and two by Haj *et al*.^25^). Further, the statistical power for all included studies under the applicable model ranged from 0.05 to 0.98. Table 3 describes the main characteristics of all the studies included in this meta-analysis, which could be divided into some diverse subgroups, regardless of some studies whose data might not be available. The genotyping method was divided into three subgroups: ‘PCR’^23^, ‘RT-PCR’^17, 22, 26, 33, 34, 37^, ‘PCR-RFLP’^21, 24, 25, 27, 31, 32^, and the diagnostic criteria were also classified into three subgroups: ‘Poser’^23, 24, 27^, ‘McDonald’^25, 26, 31^, ‘Poser & McDonald’ ^22, 34, 35, 37^. The source of control consisted of hospital-based (HB)^17, 23, 24^ and population-based (PB)^25, 26, 31^ groups. The mean age ranged from 28.8 to 45.6 years old in MS patients and from 29.4 to 54.5 years old in controls, while the percentage of females ranged from 67.50% to 71.60% in MS patients and from 49.20% to 81.90% in controls. The MS patients were mainly stratified into relapsing remitting (RR), secondary progressive (SP), and primary progressive (PP) groups, and their frequency varied from 52.9% to 91% in the RR groups, 0 to 26% in the SP groups, and 0 to 19.8% in the PP groups. In addition, the NOS results suggested that all eligible studies in this meta-analysis were of high quality because their scores were equal to or greater than six points.

**Table 2:** IL7RA genotypic distribution among MS cases and controls in the included studies.

IL7RA polymorphisms	First Author	Year	No. of Cases	No. of Controls	Cases			Controls			HWE	Power analysis*
					MM	MW	WW	MM	MW	WW		
rs3194051(8) (A > G)	Zhang	2005	667	558	54	235	378	23	230	305	0.01	0.98
	Gregory	2007	438	478	46	157	235	33	198	247	0.49	0.78
	Lundmark	2007	1785	2564	149	657	979	152	982	1430	0.33	0.99
	O'Doherty(1)	2008	208	413	18	85	105	30	163	220	0.87	0.15
	O'Doherty(2)	2008	463	531	31	178	254	36	206	289	1.00	0.05
	Akkad	2009	1279	857	102	511	666	63	346	448	0.80	0.11
	Bahlo	2009	2255	2308	152	868	1235	160	897	1251	0.96	0.07
	Kallio	2009	197	433	13	76	108	25	158	250	1.00	0.09
rs987107(6) (C > T)	Zhang	2005	528	563	53	186	289	34	228	301	0.33	0.93
	Gregory	2007	438	479	46	157	235	33	196	250	0.57	0.79
	Lundmark	2007	1779	2565	152	651	976	157	991	1417	0.35	0.99
	O'Doherty(1)	2008	207	413	17	83	107	29	160	224	1.00	0.12
	O'Doherty(2)	2008	462	527	31	178	253	35	202	290	1.00	0.05
	Jäger	2013	484	311	48	194	242	19	121	171	0.77	0.77
rs11567686(5) (A > G)	Teutsch	2003	176	176	19	79	78	18	75	83	0.86	0.12
	Broux	2010	65	33	8	29	28	2	12	19	1.00	0.48
	Hoe	2010	810	991	102	370	338	112	442	437	1.00	0.30
	Heidari	2011	100	100	18	51	31	16	47	37	0.84	0.24
	Haj	2015	202	244	49	99	54	58	102	84	0.02	0.70
rs11567685(8) (T > C)	Teutsch	2003	101	90	7	37	57	8	43	39	0.48	
	Booth	2005	363	182	28	134	201	8	84	90	0.04	
	Akkad	2009	1304	889	103	507	694	65	356	468	0.87	
	Broux	2010	65	33	6	28	31	3	14	16	1.00	
	Hoe	2010	810	991	56	313	441	71	389	531	1.00	
	Heidari	2011	100	100	9	38	53	8	44	48	0.81	
	Ibayyan	2014	200	200	4	59	137	10	78	112	0.57	
	Haj	2015	219	258	19	80	120	28	87	143	0.0127	

Note: MM: homozygous mutant genotype; MW: heterozygous mutant genotype; WW: wild genotype; NA: not available; power analysis*: rs3194051 (recessive model); rs987107 (recessive model); rs11567686 (dominant model).

**Table 3:** Main characteristics of studies included in the meta-analysis.

IL7RA polymorphisms	First Author	Year	Area	Ethnicity	Genotyping method	Diagnostic criteria	Source of controls	NOS score
rs3194051(8) (A > G)	Zhang	2005	Sweden	Caucasian	PCR	Poser	HB	7
	Gregory	2007	USA	Caucasian	RT-PCR	NA	HB	8
	Lundmark	2007	Nordic countries	Caucasian	RT-PCR	Poser&McDonald	NA	7
	O'Doherty(1)	2008	USA	Caucasian	NA	NA	NA	6
	O'Doherty(2)	2008	Northern Ireland	Caucasian	NA	NA	NA	6
	Akkad	2009	Germany	Caucasian	PCR-RFLP	Poser	NA	8
	Bahlo	2009	Australia, New Zealand	Caucasian	NA	Poser&McDonald	NA	7
	Kallio	2009	Finland	Caucasian	RT-PCR	Poser&McDonald	NA	8
rs987107(6) (C > T)	Zhang	2005	Sweden	Caucasian	PCR	Poser	HB	7
	Gregory	2007	USA	Caucasian	RT-PCR	NA	HB	8
	Lundmark	2007	Nordic countries	Caucasian	RT-PCR	Poser&McDonald	NA	7
	O'Doherty(1)	2008	USA	Caucasian	NA	NA	NA	6
	O'Doherty(2)	2008	Northern Ireland	Caucasian	NA	NA	NA	6
	Jäger	2013	Germany	Caucasian	RT-PCR	McDonald	PB	7
rs11567686(5) (A > G)	Teutsch	2003	Australia	Caucasian	PCR-RFLP	Poser	HB	6
	Broux	2010	Belgium	Caucasian	RT-PCR	NA	NA	7
	Hoe	2010	Australia	Caucasian	PCR-RFLP	NA	NA	6
	Heidari	2011	Iran	Caucasian	PCR-RFLP	McDonald	PB	6
	Haj	2015	Iran	Caucasian	PCR-RFLP	McDonald	PB	6
rs11567685(8) (T > C)	Teutsch	2003	Australia	Caucasian	PCR-RFLP	Poser	HB	6
	Booth	2005	Australia	Caucasian	RT-PCR	Poser&McDonald	NA	7
	Akkad	2009	Germany	Caucasian	PCR-RFLP	Poser	NA	8
	Broux	2010	Belgium	Caucasian	RT-PCR	NA	NA	8
	Hoe	2010	Australia	Caucasian	PCR-RFLP	NA	NA	6
	Heidari	2011	Iran	Caucasian	PCR-RFLP	McDonald	PB	6
	Ibayyan	2014	Jordan	Caucasian	PCR-RFLP	NA	NA	7
	Haj	2015	Iran	Caucasian	PCR-RFLP	McDonald	PB	6
**IL7RA polymorphisms**	**First Author**	**MS Patients**		**Controls**		**Type of MS (%)**		
		**Age (Range)**	**Female (%)**	**Age (Range)**	**Female (%)**			
rs3194051(8) (A > G)	Zhang	45.6 (15–80)	71.60%	54.5 (17–91)	49.20%	PP: 12.1		
	Gregory	31.9 (11–60)	299 (68.30%)	41.6 (21–60)	318 (66.40%)	RR:83.3, SP:12.3, PP:3.2, PR:0.7		
	Lundmark	46.4	72.00%	38.6	53.00%	RR:91, PP:9		
	O'Doherty(1)	NA	NA	NA	NA	NA		
	O'Doherty(2)	NA	NA	NA	NA	NA		
	Akkad	31.6	893 (68.00%)	47.9	425 (48.30%)	RR:55.8, SP: 24.5, PP:19.8		
	Bahlo	NA	NA	NA	NA	NA		
	Kallio	NA	NA	NA	NA	NA		
rs987107(6) (C > T)	Zhang	45.6 (15–80)	71.60%	54.5 (17–91)	49.20%	PP: 12.1		
	Gregory	31.9 (11–60)	299 (68.30%)	41.6 (21–60)	318 (66.40%)	RR:83.3, SP:12.3, PP:3.2, PR:0.7		
	Lundmark	46.4	72.00%	38.6	53.00%	RR:91, PP:9		
	O'Doherty(1)	NA	NA	NA	NA	NA		
	O'Doherty(2)	NA	NA	NA	NA	NA		
	Jäger	45	70.20%	37	50.00%	NA		
rs11567686(5) (A > G)	Teutsch	NA	NA	NA	NA	NA		
	Broux	44.7 ± 11.2 (20–68)	NA	33.2 ± 10.7 (21–55)	NA	RR:60, SP:23.1, PP:17		
	Hoe	NA	NA	NA	NA	NA		
	Heidari	28.8	78.00%	NA	68.00%	RR:65, SP:26, PP:9		
	Haj	31.8 ± 0.9	70.20%	29.4 ± 0.7	62.11%	RR:71.0, SP:17.5, PP:11.5		
rs11567685(8) (T > C)	Teutsch	NA	NA	NA	NA	NA		
	Booth	NA	NA	NA	NA	RR:52.9,SP:29.8,PP:17.3		
	Akkad	31.6	893 (68%)	47.9	425 (48.30%)	RR:55.8, SP:24.5, PP:19.8		
	Broux	44.7 ± 11.2 (20–68)	NA	33.2 ± 10.7 (21–55)	NA	RR:60, SP:23.1, PP:17		
	Hoe	NA	NA	NA	NA	NA		
	Heidari	28.8	78.00%	NA	68.00%	RR:65, SP:26, PP:9		
	Ibayyan	33.52 ± 8.92 (17–58)	135 (67.50%)	36.5 (16–64)	164 (81.90%)	RR:88, SP:10, PR:0.7, Benign:1.5		
	Haj	31.8 ± 0.9	70.20%	29.4 ± 0.7	62.11%	RR:71.0, SP:17.5, PP:11.5		

Note: HB: hospital-based; PB: population-based; NA: not available; RR: relapsing remitting; SP: secondary progressive; PP: primary progressive; PR: progressive-relapsing.

### IL7RA rs3194051 polymorphism and MS risk

**Figure 2:**
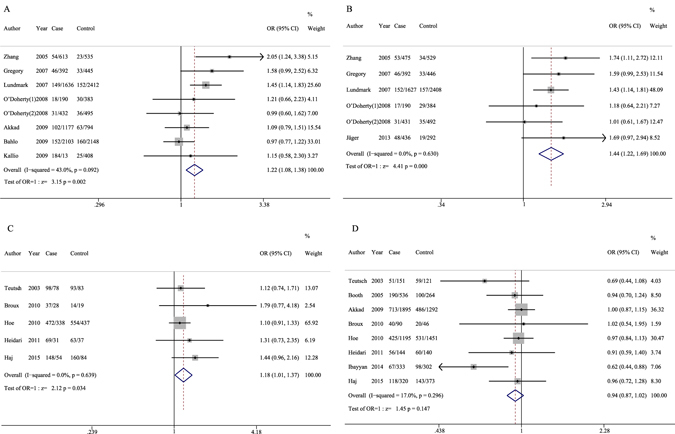
Forest plots of IL7RA polymorphisms and the risk of multiple sclerosis: (**A**): rs3194051 polymorphism under the recessive model (GG vs. GA + AA); (**B**): rs987107 polymorphism under the recessive model (TT vs. TC + CC); (**C**): rs11567686 polymorphism under the dominant model (GG + GA vs. AA); (**D**): rs11567685 polymorphism under the allelic model (C vs. T).

Eight studies containing a total of 7292 MS patients and 8142 healthy controls were included in this meta-analysis to investigate the association between the rs3194051 polymorphism and MS risk. The estimated OR1, OR2, and OR3 were 1.21 (95% CI: 1.06–1.37), 0.97 (95% CI: 0.90–1.03), and 1.17 (95% CI: 1.02–1.33), respectively, suggesting a recessive genetic effect (GG vs. GA + AA) of MS risk allele G. A fixed effect model was conducted because only moderate heterogeneity (*I* ^*2*^ = 43%) existed in the recessive model. A statistically significant association was observed between the rs3194051 SNP and the susceptibility to MS (OR = 1.22, 95% CI: 1.08–1.38) (Table 4 and Fig. 2A). In addition, the statistical power calculation based on the inclusion of sample size gave a value of 0.99, which indicated powerful evidence for the conclusion of this significant association.

**Table 4:** Meta-analysis of IL7RA polymorphisms on MS.

IL7RA variants	Genetic comparison	*I* ^*2*^(%)	Effect model	OR (95%CI)	*P*_*OR*_	Egger's test (t, p)	Statistical Power
rs3194051 (8)	GG vs. AA	31	Fixed	**1.21 (1.06, 1.37)**	**<0.01**		
	GA vs. AA	0	Fixed	0.97 (0.90, 1.03)	0.32		
	GG vs. GA	76	Random	**1.17 (1.02, 1.33)**	**0.02**		
	**GG vs. AA + GA** ^**#**^	43	Fixed	**1.22 (1.08, 1.38)**	**<0.01**	(0.61, 0.56)	0.99
rs987107 (6)	TT vs. CC	0	Fixed	**1.41 (1.20, 1.67)**	**<0.01**		
	TC vs. CC	0	Fixed	0.96 (0.88, 1.05)	0.34		
	TT vs. TC	0	Fixed	**1.48 (1.25, 1.75)**	**<0.01**		
	**TT vs. CC + TC** ^**#**^	0	Fixed	**1.44 (1.22, 1.69)**	**<0.01**	(−0.21, 0.85)	1.00
rs11567686 (5)	GG vs. AA	0	Fixed	**1.23 (0.98, 1.56)**	0.07*		
	GA vs. AA	0	Fixed	**1.16 (0.99, 1.36)**	0.06*		
	GG vs. GA	0	Fixed	1.05 (0.85, 1.30)	0.63		
	**GG + GA vs. AA** ^**#**^	0	Fixed	**1.18 (1.01, 1.37)**	**0.03**	(2.47, 0.09)	0.87
rs11567685 (8)	CC vs. TT	0	Fixed	0.96 (0.78, 1.18)	0.72	(−1.05, 0.34)	0.09
	CT vs. TT	21	Fixed	**0.90 (0.81, 1.00)**	0.06*	(−1.54, 0.18)	0.77
	CC vs. CT	0	Fixed	1.05 (0.85, 1.30)	0.63	(−0.09, 0.93)	0.08
	C vs. T	17	Fixed	0.94 (0.87, 1.02)	0.15	(−1.69, 0.14)	0.56
	CC + CT vs. TT	22	Fixed	**0.91 (0.82, 1.01)**	0.07*	(−1.70, 0.14)	0.72
	CC vs. TT + CT	0	Fixed	1.00 (0.82, 1.22)	1.00	(−0.62, 0.56)	0.05

Note: bold: significant *P*-value (<0.05); bold^*^: marginal association (0.05 < *P*-value < 0.1); ^#^Suggested model.

### IL7RA rs987107 polymorphism and MS risk

A total of 3898 MS patients and 4858 matched-controls across six studies were genotyped for the rs987107 polymorphism and MS susceptibility in this retrospective analysis. The estimated OR1, OR2, and OR3 were 1.41 (95% CI: 1.20–1.67), 0.96 (95% CI: 0.88–1.05), and 1.48 (95% CI: 1.25–1.75), respectively. These results suggested that the recessive model was also the most appropriate to be used here. We pooled the genotype data using a fixed effect model due to no between-study heterogeneity (*I* ^*2*^ = 0%). The combined data indicated that the rs987107 variant might contribute to the development of MS (OR = 1.44, 95% CI: 1.22–1.69) (Table 4 and Fig. 2B). The sample sizes of the six studies allowed full power (power = 1) to detect this OR value, which showed strong evidence for the increased association between the rs987107 polymorphism and MS risk.

### IL7RA rs11567686 polymorphism and MS risk

In this meta-analysis, we collected five related studies with a total of 1353 MS cases and 1544 controls. Pooling these studies yielded an OR1 of 1.23 (95% CI: 0.98–1.56), OR2 of 1.16 (95% CI: 0.99–1.36), and OR3 of 1.05 (95% CI: 0.85–1.30). The confidence intervals of OR1 and OR2 were slightly greater than 1which could be considered as marginally significant. Thus, the dominant pattern was regarded as the most applicable model in this case. Due to unobserved heterogeneity among studies (*I* ^*2*^ = 0%), we used the fixed-effect model to calculate the pooled OR under the dominant model. The result indicated that the rs11567686 polymorphism might confer an increased risk of MS (OR = 1.18, 95% CI: 1.01–1.37) (Table 4 and Fig. 2C) with a high statistical power value of 0.87.

### IL7RA rs11567685 polymorphism and MS risk

Eight eligible investigations with 3162 cases and 2743 normal people were included in the analysis of the association between the rs11567685 variant and susceptibility to MS. Pooling these studies generated an OR1 of 0.96 (95% CI: 0.78–1.18), OR2 of 0.90 (95% CI: 0.81–1.00), and OR3 of 1.05 (95% CI: 0.85–1.30). According to these estimates, we could not select the ideal pattern using the method of determined genetic model^29^. Therefore, six potential genetic models were performed; and the remaining three models are allelic model (OR = 0.94, 95% CI: 0.87–1.02), dominant model (OR = 0.91, 95% CI: 0.82–1.01), and recessive model (OR = 1.00, 95% CI: 0.82–1.22), respectively. The fixed effect model was conducted for all of the above statistical analyses because of no significant heterogeneity. No evidence of significant association was found under all possible genetic models, along with low statistical power (Table 4 and Fig. 2D **)**.

### Sensitivity analysis and cumulative meta-analysis

A leave-one-out sensitivity analysis showed that the pooled ORs were not significantly changed for rs3194051, rs987107 and rs11567686 variants when all the included studies, containing those three studies distracted from HWE (investigated by Zhang *et al*.^23^ and Haj *et al*.^25^), were excluded one by one. This indicated that our results were robust and reliable (data not shown).

**Figure 3:**
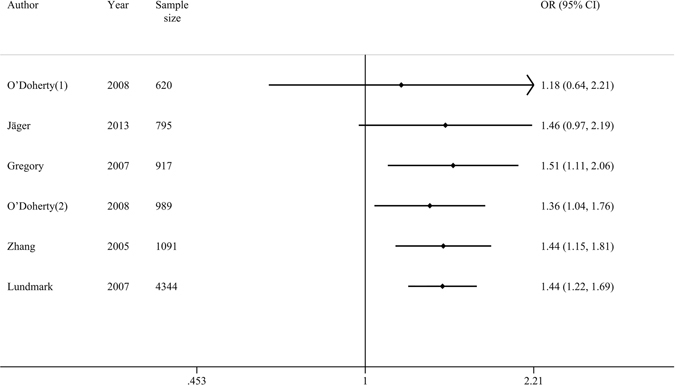
Forest plot of cumulative meta-analysis by sample size about the association between the rs987107 polymorphism and MS risk under the recessive model.

In the cumulative meta-analysis sorted by sample size, the pooled results detected a dynamic tendency of increased association between the minor variants of these three loci and the risk of MS under their most applicable genetic models, which confirmed our earlier conclusion. As an example, Fig. 3 described a tendency of increased association between the rs987107 polymorphism and MS risk in the recessive model. The combined ORs were not significantly fluctuated from accumulating each new study sample, which was also consistent with the findings of the sensitivity analysis.

### Publication bias

**Figure 4:**
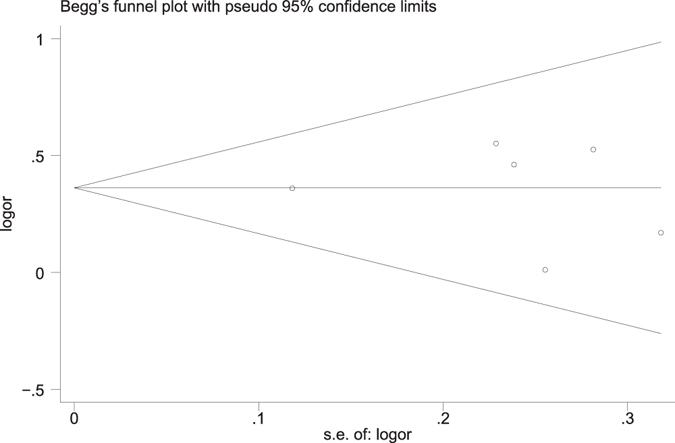
Funnel plot for the recessive model to analyze publication bias of the association of the rs987107 polymorphism with MS risk.

In the present meta-analysis publication bias was estimated by Begg’s funnel plot and Egger’s quantitative test. From the shape of funnel plots, we did not observe any asymmetric signal under all analyzed models (Fig. 4 illustrates no publication bias for the association of the rs987107 polymorphism with MS risk.). The Egger’s test also did not display any evidence of obvious publication bias for the association of these SNPs with MS risk (Table 4).

## Discussion

Multiple sclerosis, an immune mediated disease in which T cells play an important role, is the chronic inflammatory neurologic disorder of the CNS affecting young adults, especially women^38–40^. There is growing evidence that genetic factors might play vital roles in MS development. Human leukocyte antigen (HLA), for example, has been widely reported to have a strong effect on MS^11, 41, 42^. IL7RA, on the other hand, serves as the first non-HLA gene that was also determined to have an association with MS susceptibility^17, 21, 23, 43^. The present study is the first meta-analysis on the relationship between IL7RA variants and development of MS.

IL7R functions as a significant pleiotropic receptor for the signaling pathway of IL7 in autoimmune disease^44^. IL7 interacts with the IL7R common gamma chain (namely CD132) and its alpha chain (namely IL7RA or CD127), forming the signaling complex in the IL7 cascade. The IL7/IL7R interaction is vital to the survival, proliferation, and differentiation of T-cells, especially CD4+ T-cells, which exist in the inflammatory lesions of the people with multiple sclerosis^45–47^. Thus, there is no doubt that an experimental investigation of the association of polymorphisms in the IL7RA gene with MS could help us to better understand the pathogenic mechanisms and develop molecularly targeted agents for MS treatment. Previous case-control studies have reported on the association between multiple IL7RA variants and the risk of MS, but the conclusions were inconsistent because of low statistical power, small sample size, or the complex gene-gene and gene-environment interactions involved in the disease. Therefore, to evaluate the function of IL7RA polymorphisms on MS more precisely, we conducted this meta-analysis, which increased statistical power by pooling the available data from individually published studies.

We collected eight studies on the rs3194051 variant and six studies on the rs987107 polymorphism; the baseline characteristics of these studies on genetic polymorphism of these two loci shared considerable similarities. The combined results from our meta-analysis indicated that both of these IL7RA polymorphisms were associated with increasing MS risk with high statistical power; this finding is well-matched with the conclusions of two individual studies investigated by Zhang *et al*.^23^ and Lundmark *et al*.^22^. Among the other studies^17, 26, 34–36^ that suggested no obvious association between these two polymorphisms and MS risk, one involving rs3194051 and two involving rs987107 conducted by Gregory *et al*.^17^ and Jäger *et al*.^26^, respectively, was actually shown to have a significant trend of developing MS since their individual confidence intervals were just slightly across 1 (Fig. 2A,B). Moreover, the studies by O’Doherty *et al*.^36^ on both polymorphisms and the study by Kallio^34^ on the rs3194051 minor allele only suggested no relationship with MS but had relatively small sample sizes. Further, the distinct main features of cases and controls in individual studies, such as the different degrees of disease development among cases and the dissimilar genotype distributions in different geographical regions, might explain the lack of a significant association between the rs3194051 polymorphism and MS in the other two studies by Akkad *et al*.^27^ and Bahlo *et al*.^35^. Thus, our conclusions of the association of both rs3194051 and rs987107 polymorphisms with MS risk should be reliable, especially with the high statistical power calculated in this meta-analysis.

We included five studies in this meta-analysis that investigated the relationship between the rs11567686 polymorphism and MS risk. The pooled results under the dominant model concluded that the rs11567686 minor alleles were statistically significantly associated with the susceptibility to MS. This conclusion would be reliable since it was also derived from a combined odds ratio with high statistical power. Though all the included studies individually indicated that rs11567686 polymorphism was not related with increasing MS risk, two studies^25, 32^, especially the one investigated by Hoe *et al*.^32^ with the largest sample size, showed a possibility of marginal association between this polymorphism and the disease (as shown in Fig. 2C). In addition, the association was also found in the stratified subgroup of SP + PP MS patients when compared with healthy controls (*P* < 0.05) from two reports^25, 31^. Thus, the significant finding that the combined result was different from that of any of the included studies reflected the advantage of meta-analysis, by which we might properly evaluate the real genetic effect on disease development with greater statistical power through pooling all samples or synthesizing overall data available in previous studies.

In this retrospective analysis, eight studies were included to explore the effect of the rs11567685 variant on multiple sclerosis. For this association study we could not derive its most applicable genetic model. Thus, we utilized six potential genetic models to explore the association between the rs11567685 polymorphism and the predisposition to MS. Our results under all models suggested no relationship between this polymorphism and MS development, which was in agreement with the results from seven studies^24, 25, 27, 31–33, 37^. Only one study conducted by Ibayyan *et al*.^21^ indicated a association between the rs11567685 polymorphism and MS development; it is possible that the contradictory conclusion from this study might be attributed to its small sample size. Additionally, low between-study heterogeneity and a lack of publication bias obtained from this analysis further suggests the credibility of our conclusion.

There are four advantages in our study. First, meta-analysis has been recognized as an effective method to address a wide variety of clinical questions in evidence-based medicine by combining the results of multiple previously reported quantitative studies. To the best of our knowledge, this is the first meta-analysis on the issue of IL7RA variants with susceptibility to MS. Second, our meta-analysis had a relatively large sample size and strong statistical power, which helped to make the conclusion more convincing. Third, no obvious between-study heterogeneity and publication bias were observed in this retrospective analysis. Fourth, the results from both sensitivity analysis and cumulative meta-analysis confirmed the robustness of our conclusions.

The findings from the studies reviewed in this analysis should be interpreted with caution for several reasons. First, MS is a multifactorial disease and many other factors including age, gender, control of source, latitude, genotyping methods and gene-gene interactions might contribute to its susceptibility, but due to the insufficient data, we could not perform corresponding subgroup and stratified analyses to further explore in-depth reasons for MS pathogenesis. Second, our results might not be generalizable to other ethnicities because all of the included studies involved Caucasians. Third, a language bias may have existed because this meta-analysis only included English articles due to database limitations. Despite the above limitations, the present study is the first comprehensive meta-analysis with high statistical power that helps to expand our knowledge about the molecular biology and functional significance of IL7RA polymorphisms and the relationship with MS susceptibility.

Taken together, our meta-analysis indicated that IL7RA rs3194051, rs987107 and rs11567686 variants might contribute to the genetic susceptibility of MS, while the rs11567685 polymorphism had no effect on multiple sclerosis. While these results could provide a better understanding of MS pathogenesis, future well-designed studies with large sample sizes, gene-gene and gene-environment interactions are needed to confirm our present conclusions.
